# Space–Ground Remote Sensor Network for Monitoring Suspended Sediments in the Yellow River Basin

**DOI:** 10.3390/s24216888

**Published:** 2024-10-27

**Authors:** Yingzhuo Hou, Yonggang Ma, Zheng Hou, Maham Arif, Jinghu Li, Xing Ming, Xinyue Liu, Qianguo Xing

**Affiliations:** 1 CAS Key Laboratory of Coastal Environmental Processes and Ecological Remediation, Yantai Institute of Coastal Zone Research, Chinese Academy of Sciences, Yantai 264003, China; yingzhuohou@yic.ac.cn (Y.H.); jinghuli@yic.ac.cn (J.L.); 2 Shandong Key Laboratory of Coastal Environmental Processes, Yantai 264003, China; 3 University of Chinese Academy of Sciences, Beijing 100049, China; 4 Ningxia Hui Autonomous Region Hydrology and Water Resources Monitoring and Early Warning Center, Yinchuan 750001, China; 5 College of Physical Science and Technology, Hebei University, Baoding 071002, China

**Keywords:** sensor network, satellite, ground hyperspectral station, suspended sediment concentration, the Yellow River

## Abstract

The Yellow River, China’s second-largest river, is renowned for its high sediment content. In response to the potential impacts of climate change on Yellow River water resources and water environmental management, an advanced monitoring and forecasting system for water and sediment throughout the entire Yellow River basin—from its source to the sea—is urgently needed. In this paper, based on the current status of water and sediment monitoring technologies, we proposed an integrated remote sensing monitoring network that combines satellite remote sensing, drone remote sensing, and ground-based wireless automatic monitoring networks, aiming to achieve the digital monitoring of water and sediment across the entire Yellow River basin, from its upper reaches to its estuary in the Bohai Sea. By utilizing ground-based in situ hyperspectral stations for sediment source areas in the upper reaches, such as the Qingshui River basin in Ningxia, and satellite remote sensing for midstream processes in the Xiaolangdi reservoir before the flood season in 2023, as well as downstream monitoring at the Yellow River estuary, this paper demonstrates the novelty and efficiency of the space–air–ground integrated remote sensing monitoring technology.

## 1. Introduction

The Yellow River, China’s second-largest river and the fifth-largest river in the world, originates from the northern foot of the Bayan Har Mountains on the Qinghai–Tibet Plateau in the Yueguzonglie basin. It flows through nine provinces and regions, including Qinghai, Sichuan, Gansu, Ningxia, Inner Mongolia, Shanxi, Shaanxi, Henan, and Shandong, before emptying into the Bohai Sea in Kenli District, Shandong Province. The Yellow River is known as the “Mother River of China” and is an important agricultural base and economic development area in the country, but it is also prone to frequent water and sediment disasters [1].

The main causes of water and sediment disasters in the Yellow River basin include soil erosion, environmental degradation, and the excessive development of water resources, among other factors [2]. Increased summer rainfall in the upstream mountainous areas and Loess Plateau of the Yellow River leads to frequent secondary disasters like landslides and mudslides, resulting in a large influx of sediment into the river [3]. Excessive land reclamation and water resource development in the Yellow River basin have led to severe soil erosion, raising the riverbed in the middle and lower reaches and forming suspended rivers [4], posing significant potential risks.

The severity of water and sediment disasters in the Yellow River basin not only affects the livelihoods of local residents but also impacts the country’s economy and social development as a whole [5]. Utilizing mathematical models, artificial intelligence, and other methods and technologies to conduct water and sediment early warning can predict possible water and sediment disasters in the near future, issue timely warning information to relevant departments, and provide data support for evacuations and other safety measures for the people [6]; water and sediment early warning is of vital significance in safeguarding the lives, properties, and national ecological security of the people in the Yellow River basin and even at the Yellow River estuary.

China has a good foundation and experience in monitoring, early warning, and the management of water and sediment in the Yellow River [1,7]. Against the backdrop of climate change and increasing human impacts, the monitoring and early warning of water and sediment in the Yellow River require more accurate and detailed data sources to improve forecasting accuracy and enhance the scientific management. In the current era of well-developed information and Earth observation technologies, utilizing digital technology to govern the Yellow River is an inevitable trend [8,9].

The South-to-North Water Diversion Project is a major cross-basin water transfer project in China, aimed at addressing the disparity in water resources between the relatively water-rich southern regions and water-scarce northern regions [10]. The Western Route of the project is primarily tasked with mediating water resources in the western regions and achieving the cross-basin allocation of water resources [11]. It originates from Liangshan Prefecture in Sichuan Province, upstream of the Yangtze River, and ultimately transfers water resources to Gansu Province and the Ningxia Hui Autonomous Region in the Yellow River basin. The Yellow River Guxian Water Conservancy Hub Project in the middle reaches of the Yellow River has been listed as one of the key water conservancy projects in the pre-construction stage by the State Council and one of the key water conservancy projects promoted by the Ministry of Water Resources. In 2021, the Yellow River basin experienced the highest September rainfall since 1961, leading to a historically rare autumn flood in the middle and lower reaches of the Yellow River [12,13], with the water level at Xiaolangdi reservoir reaching its highest level since construction at 273.5 m; the sediments and nutrients from the Yellow River basin were expected to bring huge impacts on the marine ecosystem and aquaculture [14,15,16,17].

The main components of water and sediment monitoring in the Yellow River, similar to other rivers, include hydrological, sedimentological, meteorological, and geomorphological aspects [5,18]. Hydrological observation involves the real-time monitoring of river water levels, flow rates, and other hydrological elements by setting up hydrological stations, installing instruments like water level gauges and flow meters in the river, and collecting data regularly to grasp the hydrological conditions of the river in a timely manner. Sediment observation entails monitoring suspended sediment concentration (SSC), sediment constituents, etc., by collecting water samples at sediment stations in the river channel and conducting laboratory analyses on sediment samples to obtain information on sediment content and particle size [19,20]. Meteorological monitoring involves setting up meteorological stations to monitor meteorological elements such as temperature, precipitation, and wind direction to assess the impact of weather changes on water and sediment in the river [21]. Geomorphological monitoring evaluates the influence of terrain and vegetation in the basin on water and sediment yield, as well as potential events like debris flows and flood-prone areas, using on-site and remote sensing methods [22,23].

**Figure 1:**
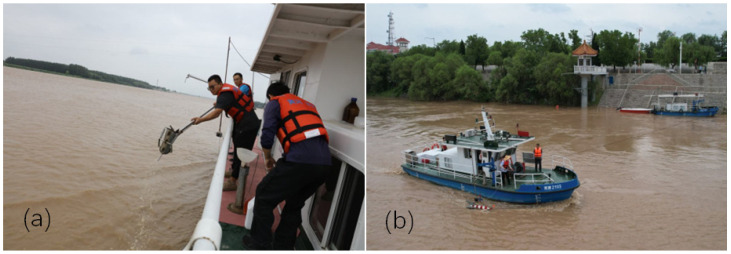
The traditional regular sampling and measurements of water and sediments in the Yellow River. (**a**) On-site water sampling operations conducted by staff at the Lijin hydrological station, and (**b**) On-site flow velocity measurement operations by staff at the Luokou hydrological station during the water and sediment regulation period before the Yellow River flood in 2023. (Photo credits Xinhua News, 12 July 2023 16:43, https://www.news.cn/2023-07/12/c_1129745575.htm, accessed on 25 April 2024).

The technical means of water and sediment monitoring in the Yellow River mainly include (1) Traditional manual monitoring: Collecting water samples on-site (as shown in Figure 1) and conducting further analysis in the laboratory using instruments; (2) Sensor monitoring: Various sensor devices, such as water quality sensors and sediment sensors, are widely used in modern hydrological monitoring [24]. They can be directly installed in the river to monitor water quality and sediment conditions in real-time and to transmit monitoring data to the monitoring center through data transmission channels, with the characteristics of unmanned automatic operation; (3) Satellite remote sensing monitoring: Utilizing satellite remote sensing allows for the comprehensive and rapid monitoring of river hydrology, sediment, and terrain features [25,26]. High-resolution image data can be obtained through satellite sensors, combined with remote sensing image interpretation and digital image processing techniques to extract and analyze information on water and sediment in the river.

Given the changing climate and hydrological situations and the construction of major projects on the Yellow River [27], the development of relevant technologies for the digital governance of the Yellow River is imperative. Building on the current status of water and sediment monitoring technology, this article proposes an integrated remote sensing monitoring network for water and sediment covering the sky, land, and sea using satellite remote sensing, drone remote sensing, and ground-based wireless automatic monitoring networks, aiming to achieve the digital monitoring of water and sediment throughout the entire Yellow River basin from the upper reaches to the Bohai Sea estuary. This article introduces the construction of in situ hyperspectral stations from the upstream sediment source to the Qingshui River section and remote sensing monitoring, as well as some multi-source satellite monitoring demonstrations of water and sediment transport processes in the downstream areas.

## 2. Data and Methods

### 2.1. Study Area

**Figure 2:**
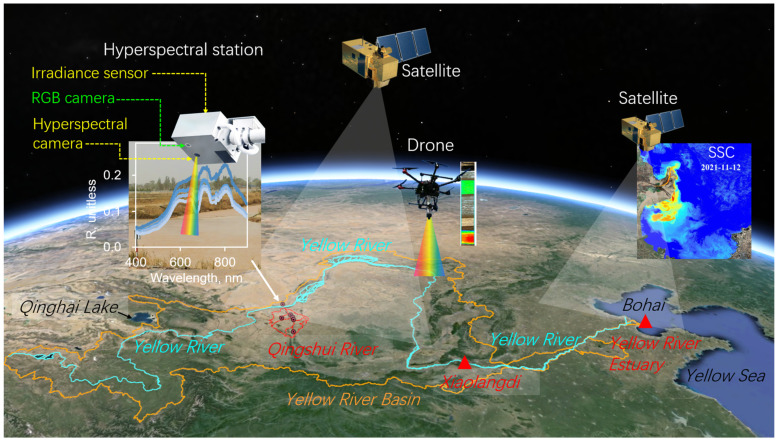
Schematic diagram of the integrated remote sensing monitoring network for water and sediment in the Yellow River basin.

Using ground-based and drone-based hyperspectral technology, researchers have achieved high prediction accuracy in monitoring complex optical and non-optical water quality parameters [28,29,30,31]. The integrated monitoring of air, space, land, and sea has been applied in monitoring and evaluating water and sediment transport, transboundary disasters, etc., with good results [32,33]. Figure 2 shows the schematic diagram of the integrated remote sensing monitoring network for water and sediment in the Yellow River basin. The upper and middle reaches of the Yellow River are mostly sediment production areas, such as the Loess Plateau, focusing on monitoring and predicting the processes of soil erosion and sediment production, while the middle and lower reaches of the river, especially the Hukou section, pay attention to the accumulation and flooding of the river channel. The lower reaches, including the Yellow River delta and estuary, focus on the ecological replenishment of wetlands, the accumulation of sediment in the estuary, and the impact of suspended sediment on water masses and the marine ecosystem [17,34,35,36]. This study presents an integrated remote sensing monitoring network for water and sediment, using the Qingshui River basin in Ningxia, the Xiaolangdi reservoir, and the adjacent Yellow River estuary as case studies.

### 2.2. Data

#### 2.2.1. Measured Spectra and Suspended Sediment Concentration

In this study, water reflectance spectral data were acquired using a ZK-UVIR-I in situ hyperspectral spectral water quality monitoring instrument. The spectral range of the instrument is 350–950 nm, with data from the commonly used range of 400–900 nm selected for analysis. The spectral resolution is 0.5 nm. Data collection took place from 26 August 2022 to 26 September 2022 at the Wangtuan station in the Qingshui River basin, Ningxia, China. The actual suspended sediment concentration (SSC) in the water body was determined through manual field sampling and laboratory filtration. Water sampling was conducted using the "single-line waterfront and one-point surface" approach, with sampling times synchronized with the acquisition of hyperspectral data. Regarding the sampling frequency, when the river flow was high, at least seven samples were collected per day, while under normal conditions, a minimum of one sample per day was taken. The determination of SSC using the filtration method involves the following steps: (1) A specific volume of water sample is collected using a graduated cylinder and allowed to settle; (2) The supernatant is decanted, and the sediment is poured onto filter paper, allowing the water to percolate; (3) The sediment wrapped in filter paper is dried in an oven; (4) The dry sediment is weighed using a balance with a precision of 0.001 g; (5) The SSC is calculated as the ratio of the dry sediment mass to the volume of the water sample.

#### 2.2.2. Satellite Imagery

The Digital Elevation Model (DEM) data used for analyzing the water and sediment conditions in the Qingshui River basin were obtained from the Geospatial Data Cloud site (GSCloud, https://www.gscloud.cn/, accessed on 10 May 2024), with a spatial resolution of 30 m. The normalized difference vegetation index (NDVI) data were derived from the median NDVI values of Landsat-8 imagery acquired between April and August 2023, with a spatial resolution of 30 m. The Landsat-8 imagery was downloaded from the U.S. Geological Survey (USGS, https://earthexplorer.usgs.gov/, accessed on 10 May 2024). For analyzing the suspended sediment concentration (SSC) in the Xiaolangdi reservoir and the adjacent Yellow River estuary, a total of 12 satellite images were used, including CB04A, HJ2A/B, and CM1 imagery. SSC inversion was performed on 11 of the 12 downloaded images (excluding the CM1 imagery) obtained from the China Centre for Resources Satellite Data and Application (CRESDA, https://data.cresda.cn/, accessed on 25 June 2024), with all images resampled to a spatial resolution of 16 m.

Additionally, the hydrological station measurement data, as well as the sediment transport, discharge, and sediment concentration data for the Yellow River, are sourced from the Yellow River Daily Hydrological Bulletin, Yellow River Sediment Bulletin, Yellow River Water Resources Bulletin, and the Yellow River Hydrological Reports. These data are available for download from the official website of the Yellow River Conservancy Commission of the Ministry of Water Resources (http://www.yrcc.gov.cn/, accessed on 25 June 2024).

### 2.3. Methods

For the Wangtuan station, after quality control and temporal alignment, 47 paired measurements of water spectral data and SSC were collected for model development. The construction of the SSC model involves the following steps: (1) Resampling the raw spectra to 10 nm to ensure applicability to hyperspectral data acquired from drones or satellites; (2) Using single-band reflectance, band differences, and band ratios as input variables (X), and SSC as the output variable (Y); (3) Fitting the data after transforming X and Y into a linear relationship; (4) Selecting the optimal model based on the coefficient of determination (R^2^). Among them, the band difference model, SSC = 2 × 10^−5^ (R690−R680)^−2.512^ (R690 represents the reflectance at 690 nm) yielded the best performance, with an R^2^ value of 0.67. Therefore, this model was used for temporal SSC monitoring at the Wangtuan station [37].

For monitoring SSC in the Xiaolangdi reservoir and the adjacent coastal areas of the Yellow River estuary, this study primarily relies on multispectral satellite imagery. We adopted the SSC monitoring model developed by Xing et al. (2014) [38] using HJ satellite data for the Yellow River estuary. The model is defined as SSC = 2.3224 exp(59.445 × R830), with an R^2^ value of 0.93, demonstrating its suitability for SSC monitoring in highly turbid waters.

## 3. Results and Analysis

### 3.1. Hyperspectral Monitoring of Water and Sediment in the Qingshui River

The Qingshui River is a tributary on the right bank of the Yellow River, flowing through the Ningxia Hui Autonomous Region. The Qingshui River basin in Ningxia belongs to a temperate continental climate, with cold winters and hot summers, significant day–night temperature differences, and an area of approximately 16,000 square kilometers, with a water area of about 53,000 hectares, mainly including the Qingshui River itself and its tributaries, lakes, and other water bodies. It is an important part of the water resources in Ningxia and the Yellow River basin, with abundant natural resources and a unique ecological environment. The water resources of the Qingshui River basin supply local needs for agricultural irrigation, domestic water use, and industrial water use. The terrain of the Qingshui River basin in Ningxia is characterized by significant fluctuations, mainly mountainous and hilly areas with narrow river valleys. Precipitation is concentrated in the summer, often with intense rainfall, leading to flash floods [39].

**Figure 3:**
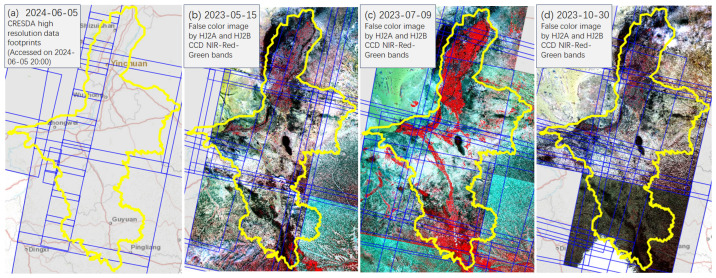
High-resolution satellite remote sensing data for the upper reaches of the Yellow River in Ningxia: (**a**) Daily domestic high-resolution optical satellite from CRESDA, (**b**–**d**) vegetation changes in May, July, and October (vegetation areas are shown in red, clear water areas in dark color). The blue lines represent the ground footprints of the image distribution, the yellow line indicates the spatial extent of Ningxia, and the red lines mark the roads in the base map. “2024-06-05” represents the date 5 June 2024, and the same applies to the other figures.

**Figure 4:**
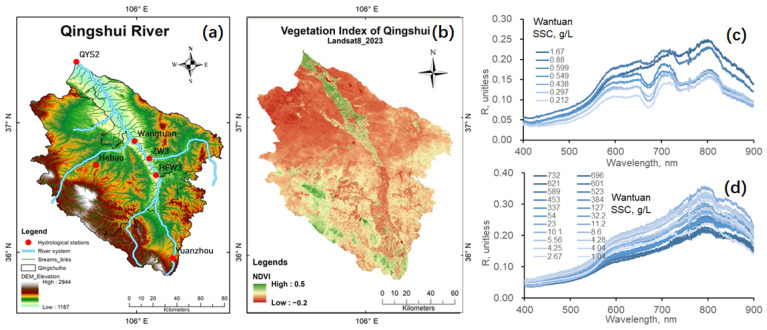
(**a**) DEM of the Qingshui River basin, (**b**) vegetation in the Qingshui River basin, (**c**,**d**) typical spectra of high-concentration sediment at the Wangtuan spectral station in the Qingshui River basin.

As shown in Figure 3, high-resolution imagery can be used to monitor changes in land cover in the Qingshui River basin. The Normalized Difference Vegetation Index (NDVI) is an index used to reflect the degree of vegetation cover and growth conditions on the ground, with values ranging from −1 to 1. A higher value indicates better vegetation cover (Figure 4b). In the Qingshui River basin, the NDVI index can be used to analyze trends in vegetation changes, including vegetation cover, seasonal variations, and desertification levels, and to assess the erosion modulus of the basin soil [40]. By combining DEM (Figure 4a) and precipitation forecasts, the erosion status of the soil–water system at different times can be assessed [41], and the water volume and sediment concentration in the river can be predicted.

Real measured data from 12 hydrological stations in the Loess Plateau region show that sediment concentrations can range from as low as 0.41 mg/L to as high as 1080 g/L; satellite remote sensing results indicate that, in the Yellow River channel affected by the Loess Plateau, the sediment concentration in summer and autumn reaches 2.998 g/L, while, in winter and spring, it decreases to as low as 1.126 g/L [26]. The Qingshui River basin is part of the Loess Plateau, and, as a tributary, its narrow river channel makes it difficult to accurately estimate sediment concentrations using satellite remote sensing data with a resolution of 30 m. In situ hyperspectral monitoring can be used to monitor sediment concentrations, as shown in conventional hydrological stations and the Wangtuan station in the Qingshui River basin, with sediment concentrations reaching as high as 732 g/L (Figure 4c,d). The spectral data recorded at this station not only reflect changes in sediment concentrations but also capture variations in organic matter such as algae in the Qingshui River, as shown in Figure 4c.

**Figure 5:**
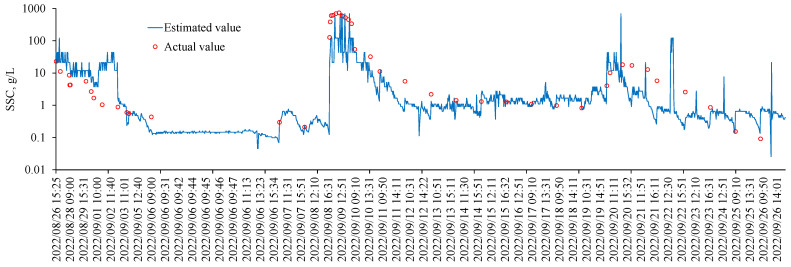
Time series of suspended sediments concentrations at the Wangtuan hyperspectral station: 26 August 2022 to 26 September 2022. The red circles are the actual SSC measured by discrete in situ water sampling; The blue lines are the SSC retrieved from the hyperspectral reflectance in the continuous measurements.

As shown in Figure 5, during the period from 26 August 2022 to 26 September 2022, the daytime sediment concentration at the Wangtuan station can be monitored in real time through hyperspectral reflectance ratios, with a time resolution better than 10 min. The sediment concentration obtained through this non-contact hyperspectral real-time inversion is consistent with the synchronously measured sediment concentration obtained through discrete on-site manual sampling (as shown in Figure 5). The discrepancy between some of the estimated SSC values and the actual values is primarily due to errors in the SSC retrieval model, measurement errors in the actual SSC, and the time discrepancy between the collection of spectral data and water samples. Based on the times of several high-concentration sediment peaks detected by the spectral station, such as on 26 August 2022, 9 September 2022, and 20 September 2022, these are consistent with the precipitation times during the same period in the region, indicating that local precipitation in the Qingshui River basin can lead to rapid responses and changes in sediment concentration.

### 3.2. Satellite Remote Sensing for Water and Sediment Regulation Before the 2023 Flood Season of the Yellow River

The majority of sediment in the middle and lower reaches of the Yellow River comes from the Loess Plateau. The accumulation of sediment poses a significant risk, and intermediate reservoirs such as Xiaolangdi face ongoing sediment deposition issues. From the implementation of water and sediment regulations in 2002 until all the water and sediment reaching the sea by 2023, approximately 3.29 billion tons of sediment have been transported to the sea. The average depth of the main channel downstream has decreased by 3.10 m, and the minimum flow capacity of the main channel has increased from 1800 m^3^/s before the flood of 2002 to around 5000 m^3^/s now, reducing the likelihood of mid to small floodplain inundation downstream. Over the 22 years of water and sediment regulation, Xiaolangdi reservoir has discharged a cumulative total of 2.705 billion tons of sediment.

**Figure 6:**
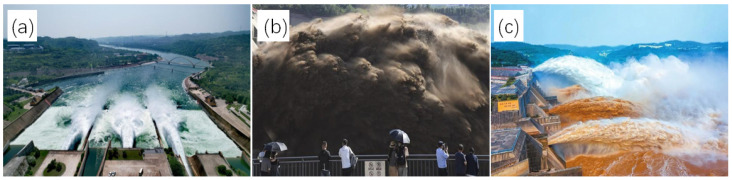
Different water colors of the Yellow River with varying SSC at the Xiaolangdi reservoir. (**a**) Clear water released from the Xiaolangdi reservoir during the primary water and sediment regulation phase before the flood in 2023. (Xinhua News, 12 July 2023 16:43, https://www.news.cn/2023-07/12/c_1129745575.htm, accessed on 5 June 2024); (**b**) Sediment was discharged from the Xiaolangdi reservoir on the Yellow River in Jiyuan, Henan on 7 July 2023. (Pengpai News, 11 July 2023 17:48, https://www.thepaper.cn/newsDetail_forward_23809045, accessed on 5 June 2024); (**c**) Xiaolangdi flood discharge on 2 October 2021. (Henan Daily, https://baijiahao.baidu.com/s?id=1713561072209768428&wfr=spider&for=pc, accessed on 5 June 2024).

**Figure 7:**
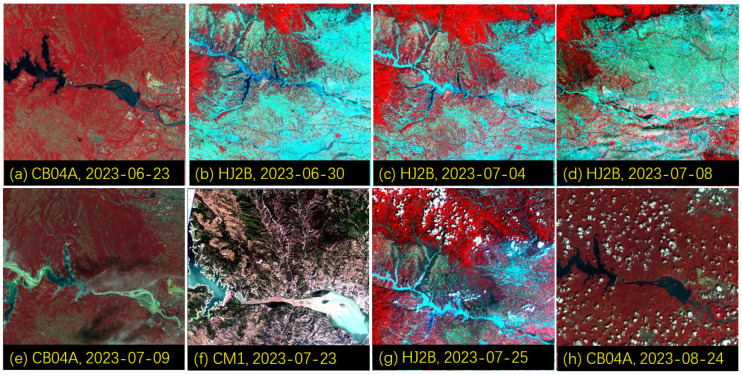
The changes in the water and sediment status of the Xiaolangdi reservoir and downstream areas during the water and sediment regulation period of the Yellow River before the flood in 2023 (from 21 June 2023 to 11 July 2023). Except for Figure (**f**), which is a true-color composite image, all other figures are false-color composites (the false-color image is composed of the NIR, Red, and Green bands; in the false-color image, vegetation areas are shown in red, clear water areas in dark color, and water bodies with high sediment content in brighter colors; the same applies to the following figures).

**Figure 8:**
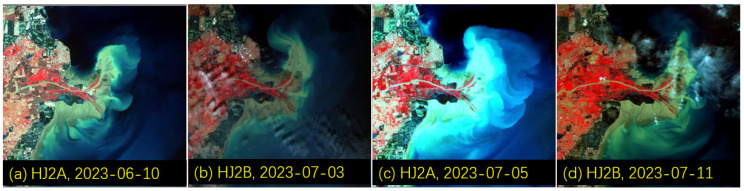
The changes in the water and sediment status of the Yellow River estuary and adjacent water bodies during the water and sediment regulation period of the Yellow River before the flood in 2023 (from 21 June 2023 to 11 July 2023). Both the HJ2A and HJ2B remote sensing images in the figure are false-color composites and have the same spatial resolution of 16 m. The color difference descriptions are the same as those in Figure 7.

The water colors in the Yellow River varied sharply in different seasons and different phases of water and sediment regulation (as shown in Figure 6). In Figure 7 and Figure 8, based on high-resolution satellite remote sensing images, the water and sediment changes in the Xiaolangdi reservoir and the Yellow River estuary during the water and sediment regulation period before the 2023 flood are displayed. This water and sediment regulation started on 21 June and concluded on 11 July at 8 a.m. through coordinated water release from reservoirs such as Wanjiazhai, Sanmenxia, and Xiaolangdi to implement the Yellow River water and sediment regulation before the 2023 flood, with all the water and sediment reaching the sea by around July 16. The Xiaolangdi and Sanmenxia reservoirs discharged 125 million tons and 40 million tons of sediment, respectively, with the volume of water reaching the sea and the volume of sediment reaching the sea being 4.259 billion cubic meters and 27 million tons, respectively. As shown in Figure 7a,b, during the clear water discharge phase (Figure 6a), the sediment concentration in the water released from Xiaolangdi reservoir was low. On 4 July at 22:00, sediment discharge began from Xiaolangdi reservoir (Figure 6c), and, by 10:30 a.m. on 7 July, the maximum sediment concentration reached 417 g/L. Figure 7d,e shows extremely high sediment levels in the downstream channel of Xiaolangdi on 8 and 9 July.

Figure 8 displays the changes in water and sediment at the Yellow River estuary during the same period. The maximum flow at the Lijin hydrological station was 3730 m^3^/s on 3 July 2023. Under the influence of the high tide, the extent of the turbid water mass at the Yellow River estuary was largest on 5 July. The SSC at the Lijin hydrological station reached a maximum of 11.80 g/L on 11 July at 8 a.m., but the impact was not significant on the same day, as shown in Figure 8d. These results highlight the complexity of water volume and sediment concentration affecting the estuarine water body.

**Figure 9:**
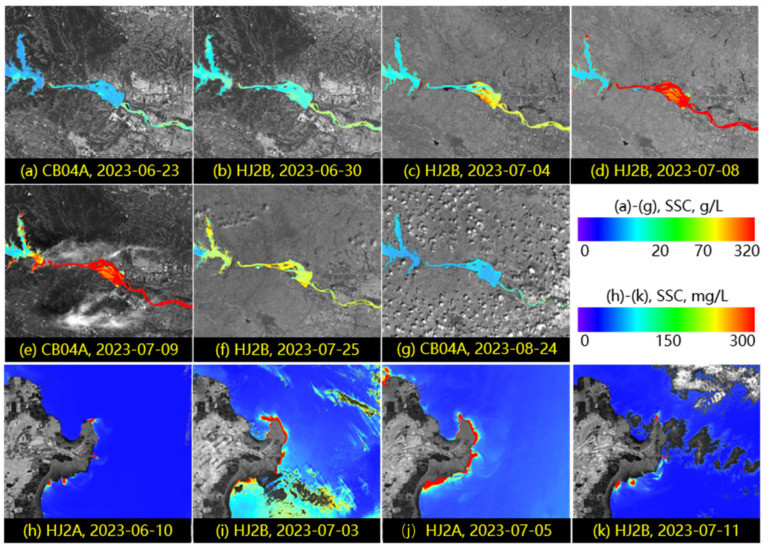
The SSC during the water and sediment regulation period of the Yellow River before the flood in 2023 (from 21 June 2023 to 11 July 2023). (**a**–**g**), the SSC at Xiaolangdi reservoir and downstream areas; (**h**–**k**) the SSC at the Yellow River estuary.

According to the method proposed by Xing et al. (2014) [38] for Chinese environmental monitoring satellites, the SSC was quantitatively retrieved for the satellite images (Figure 7 and Figure 8) over the Xiaolangdi reservoir and the Yellow River estuary. The temporal and spatial changes in SSC are shown in Figure 9, and a highly dynamic range in SSC from several milligrams per liter to hundreds of grams per liter was also captured by satellite remote sensing over the Yellow River, as captured by an in situ spectral station over the Qingshui River (Figure 5).

## 4. Discussion

### 4.1. Strengths and Limitations of Space-Air-Ground Integrated Remote Sensor Network

In response to the diversity of water and sediment monitoring content and the inadequacy of traditional monitoring methods, the integrated remote sensing monitoring network supported by advanced ground observation technology can provide efficient monitoring methods for water and sediment in the Yellow River. Its main technologies include satellite remote sensing, drone remote sensing, and ground-based wireless automatic monitoring networks, with the following main advantages and disadvantages.

(1) Satellite remote sensing: Virtual high-resolution optical satellite constellations such as HJ, GF, Landsat, Sentinel-2, and PRISMA from China, the United States, and the European Union can achieve high-frequency observations multiple times per day, providing synchronous water and sediment topographic information over a large spatial range and first-hand information for disaster areas with transportation difficulties [34,36,38]. Its shortcomings include the inability to work at night; in cloudy and rainy weather conditions, cloud cover over the target area renders optical remote sensing satellite monitoring ineffective. Microwave can work in all weather conditions and can be used to identify water bodies but cannot calculate sediment concentration within them.

(2) Drone remote sensing: With flexible maneuverability, route-planning adjustments, and rapid response capabilities [42], drones and networked observations can provide ultra-high-resolution spatial information on water and sediment disasters; high-endurance drones can provide water and sediment information over a large spatial range. However, drones cannot provide water and sediment information at night; the accuracy of drone remote sensing is greatly affected by cloudy and rainy weather, and corresponding inversion algorithms need further development. Additionally, the observation area and flight paths of drones are subject to local airspace regulations. The flight altitude and speed must be adjusted based on the specific conditions of the observation site to ensure the safety of both personnel and the drone.

(3) Ground-based in situ hyperspectral sensing network: Deploying a wireless automatic monitoring network at critical water body sections can provide real-time water and sediment information during the day, enabling real-time field data for the routine monitoring of water and sediment and disaster warning. Conventional contact automatic monitoring stations suffer from high losses, instability, and maintenance difficulties. Automatic in situ hyperspectral sensors based on near-ground remote sensing principles have the advantages of non-contact, environmental friendliness, and low maintenance costs, but they are easily affected by changes in lighting conditions and cannot operate at night [28,29]. Further research is needed on corresponding technologies, including active light source monitoring for night operations.

### 4.2. Prospects for Automatic In Situ Hyperspectral Sensing Network

The automatic in situ hyperspectral sensing network is a technology developed based on the on-site hyperspectral monitoring technology of water components and water quality, combined with automation and wireless communication technology [43,44,45,46]. It is not only applied in water quality monitoring [28,29,47], but also plays an important role in satellite remote sensing and drone remote sensing calibration [47,48,49]. It is an important infrastructure for integrated remote sensing monitoring networks that connect space, air, and ground.

Using an automatic in situ hyperspectral sensing network to monitor water and sediment has the following prominent characteristics: (1) Non-contact: The in situ non-contact spectral method does not require direct contact with the water. It can obtain the spectral information of the water from a distance through remote sensing technology, avoiding the need for direct sampling in traditional monitoring methods, reducing interference with water samples, and helping to maintain the original characteristics of the water; (2) Real-time: The in situ non-contact spectral method can achieve remote, real-time monitoring. Through spectral instruments, it can, in real-time, obtain the spectral characteristic parameters of the water, promptly reflecting changes in composition and concentration in the water, and providing data support for rapid response to water changes; (3) Efficiency: Compared to traditional sampling and laboratory analysis, the in situ non-contact spectral method for monitoring water and sediment is more efficient, reducing sampling, transportation, pre-processing, and other links, shortening the monitoring period, and improving monitoring efficiency [50]; (4) Environmental friendliness: The in situ non-contact spectral method does not require a large number of chemical reagents and laboratory analysis equipment, reducing environmental pollution and resource waste, in line with the environmental requirements of energy conservation and emission reduction.

In summary, using the in situ non-contact spectral method to monitor water and sediment has significant advantages. It can improve monitoring efficiency, reduce monitoring costs, and achieve remote, real-time, and comprehensive monitoring. It provides new technical means and ideas for water and sediment monitoring and water resource management.

## 5. Conclusions and Prospects

With the globally increasing demand for the utilization of water resources and the mitigation of flooding hazards, advanced systems for monitoring water and sediments in efficient and environmentally friendly ways are essential. A space–ground remote sensing network, combining satellites, drones, and ground-based hyperspectral stations, is proposed for monitoring the Yellow River watershed. As the recent demonstrations show in this work, this system would be expected to provide fine sustainable water and sediment monitoring and management applications for the Yellow River basin from the upper-reach small watersheds, main river channel, and reservoirs to its estuary in a continuous spatiotemporal dynamic manner.

Through the integrated remote sensing monitoring network, not only can water and sediment be monitored and warned, but the water quality monitoring of the Yellow River basin and estuarine waters can also be conducted in the future. Parameters such as chlorophyll concentration, algae blooms, transparency, chemical oxygen demand (COD), and other parameters can be retrieved, and the assessment of water body functions can be performed to enable more rational water resources development, utilization, and management. And it could be expected that more sensor instruments and algorithms with more reliable performance will be developed with more applications of the space–ground remote sensing network in future.

## Data Availability

The raw data supporting the conclusions of this article will be made available by the authors on request.
